# Plastid genome data provide new insights into the dynamic evolution of the tribe Ampelopsideae (Vitaceae)

**DOI:** 10.1186/s12864-024-10149-w

**Published:** 2024-03-05

**Authors:** Lei Zhang, Ying Meng, Da Wang, Guan-Hao He, Jun-Ming Zhang, Jun Wen, Ze-Long Nie

**Affiliations:** 1 Hunan Provincial key Laboratory of Ecological Conservation and Sustainable Utilization of Wulingshan Resources, College of Biology and Environmental Sciences, Jishou University, Jishou, Hunan 416000 China; 2 Department of Botany, National Museum of Natural History, Smithsonian Institution, Washington, DC 20013-7012 USA

**Keywords:** Ampelopsideae, Plastid genome, Comparative analysis, Phylogenomics

## Abstract

**Background:**

Ampelopsideae J. Wen & Z.L. Nie is a small-sized tribe of Vitaceae Juss., including ca. 47 species from four genera showing a disjunct distribution worldwide across all the continents except Antarctica. There are numerous species from the tribe that are commonly used as medicinal plants with immune-modulating, antimicrobial, and anti-hypertensive properties. The tribe is usually recognized into three clades, i.e., *Ampelopsis* Michx., *Nekemias* Raf., and the Southern Hemisphere clade. However, the relationships of the three clades differ greatly between the nuclear and the plastid topologies. There has been limited exploration of the chloroplast phylogenetic relationships within Ampelopsideae, and studies on the chloroplast genome structure of this tribe are only available for a few individuals. In this study, we aimed to investigate the evolutionary characteristics of plastid genomes of the tribe, including their genome structure and evolutionary insights.

**Results:**

We sequenced, assembled, and annotated plastid genomes of 36 species from the tribe and related taxa in the family. Three main clades were recognized within Ampelopsideae, corresponding to *Ampelopsis*, *Nekemias*, and the Southern Hemisphere lineage, respectively, and all with 100% bootstrap supports. The genome sequences and content of the tribe are highly conserved. However, comparative analyses suggested that the plastomes of *Nekemias* demonstrate a contraction in the large single copy region and an expansion in the inverted repeat region, and possess a high number of forward and palindromic repeat sequences distinct from both *Ampelopsis* and the Southern Hemisphere taxa.

**Conclusions:**

Our results highlighted plastome variations in genome length, expansion or contraction of the inverted repeat region, codon usage bias, and repeat sequences, are corresponding to the three lineages of the tribe, which probably faced with different environmental selection pressures and evolutionary history. This study provides valuable insights into understanding the evolutionary patterns of plastid genomes within the Ampelopsideae of Vitaceae.

**Supplementary Information:**

The online version contains supplementary material available at 10.1186/s12864-024-10149-w.

## Introduction

Ampelopsideae is a small-sized tribe and the first diverged lineage of the grape family of Vitaceae, including ca. 47 species from four genera showing a disjunct distribution worldwide across all the continents except Antarctica [1, 2]. Members of the tribe are morphologically characterized by the inflorescence mostly five-parted with a cup-shaped disc, which is slightly lobed and adnate proximally to the base of the ovary while being free distally [2]. The tribe contains many species that can be used medicinally, such as *Ampelopsis delavayana* Planch. ex Franch. and *Ampelopsis japonica* (Thunb.) Makino. possess immunomodulatory and antimicrobial activity, and treats hypertension function [3–5].

Three lineages are usually recognized within Ampelopsideae, i.e., *Ampelopsis*, *Nekemias*, and the Southern Hemisphere clade [1, 6, 7]. However, the relationships of the three clades differ greatly between the nuclear and the plastid topologies [1, 7, 8]. Nuclear data indicate that *Ampelopsis* is the first diverged lineage, sister to a clade including *Nekemias* and the clade composed of *Rhoicissus* Planch. and *Clematicissus* Planch. from the Southern Hemisphere [7, 8]. Nonetheless, the plastid tree proposed *Nekemias* as the first diverged lineage within the tribe [7]. *Nekemias* is similar in distribution as *Ampelopsis*, with most species occurring in East Asia and only a few in North America [1]. Because of their distributional and morphological similarities, taxonomists traditionally placed *Nekemias* in *Ampelopsis* [9]. Both nuclear and plastid gene data support the embedding of taxa from Southern Hemisphere into the traditional *Ampelopsis* [1, 8].

Plastids genomes in angiosperms are highly conserved with similar structure, gene sequences and organization, with length between 120 to 160 kb in size [10]. They comprise a large single copy region (LSC; 80–90 kb), a small single copy region (SSC; 16–27 kb), and two inverted repeat regions (IRs) of approximately 20–28 kb each [10]. Because of their conserved structure, low occurrence of recombination, and primarily uniparental inheritance, plastid sequences have been extensively employed as preferred markers for plant phylogenetics and evolution [1, 11–14]. Although the plastid genome is usually conserved [15, 16], structural rearrangements, gene loss, IR expansions, and inversions occur in certain lineages and provide useful insights into phylogenetic evolution in plants [17, 18]. For example, plastid genome sequences have been utilized for DNA barcoding, phylogenetic, transplastomic and population questions [19–24].

Recent advances in genomic sequencing have led to the availability of complete plastid genomes, which provide more comprehensive information for phylogenetic studies. Although previous studies have investigated the chloroplast genomes of individual or a few species in the grape family, including some taxa from Ampelopsideae, such as *A. delavayana*, *A. japonica* and *Nekemias cantoniensis* (Hook. & Arn.) J. Wen & Z.L. Nie [25, 26], expanding the sampling of the tribe would be beneficial for understanding the plastid structural evolution within Ampelopsideae.

In this study, we aimed to newly sequence and assemble plastid genomes of a total of 36 species from Ampelopsideae and closely related taxa, in order to investigate the evolutionary characteristics of plastid genomes of the tribe, including their genome structure and evolutionary insights. We hypothesized that a broad sampling of the tribe would provide a more comprehensive understanding of its plastome evolutionary pattern. Our results may also provide insights into the evolution of other taxa of the economically highly significant grape family and inform future research on their molecular, morphological, geographic, and ecological diversification.

## Materials and methods

### Plant materials, DNA extraction and sequencing

In this study, we sampled a total of 36 accessions, including 30 individuals representing 22 species from Ampelopsideae, plus 6 from other genera of the family (i.e., *Parthenocissus* Planch., *Cissus* L., *Cayratia* Juss. and *Pseudocayratia* J. Wen, L.M. Lu & Z.D. Chen). All the samples were newly sequenced except that two species from *Ampelopsis* were obtained from NCBI (MK574541 and MK574542). In accordance with previous researches [1, 7, 8], *Leea guineensis* G. Don. (MW592489), a species from Leeaceae Dumort., the sister family of Vitaceae, was utilized as a remote outgroup for reconstructing the phylogenetic tree. Information on the plant material (collection localities and voucher specimen numbers) and the associated GenBank accessions are listed in Supplementary Table 1.

A modified CTAB method was used to extract total DNA from either silica gel-dried leaves or plant specimens [27, 28]. Extracted DNAs were quantified on a Qubit 4.0 fluorometer (Thermo Fisher Scientific) using a high-sensitivity kit and then sheared to a target size ca. 300–500 bp by sonication (QSonica Q800RS). DNA libraries were generated with the NEBNext Ultra DNA Kit following the manufacturer’s protocol. The libraries were then sequenced on an Illumina HiSeq 4000 platform using a 150 paired-end protocol.

### Data assembly and annotation

Clean raw data were used to assemble complete plastid genome sequences by the program GetOrganelle [29], and then annotated using GeSeq (https://chlorobox.mpimpgolm.mpg.de/geseq.html) [30]. The obtained sequences were checked and manually adjusted in the program Geneious-9.0.2 using *Ampelopsis humulifolia* Bunge. as a reference. Finally, all the newly sequenced plastid genomes were uploaded to NCBI (Supplementary Table 1). Additionally, plastid genomic maps were generated from https://chlorobox.mpimp-golm.mpg.de/OGDraw.html [31].

### Phylogenetic analysis

The completed plastids genome sequences were aligned using MAFFT 7.427 [32]. Phylogenetic analysis was conducted based on maximum likelihood (ML) analysis using the GTRGAMMA nucleotide substitution model with the default parameters in RAxML 7.2.6 [33]. RAxML allows for only a single evolutionary model in partitioned analyses, which was selected according to PartitionFinder2 results. Bootstrap supports (BS) were estimated using a rapid bootstrapping algorithm and 1000 replicates in RAxML.

### Plastome comparative analyses

The simple sequence repeats (SSR) were detected by MISA (https://webblast.ipk-gatersleben.de/misa/), with parameters set to ten, five, and four repeats for mononucleotide, dinucleotide, and trinucleotide [34]. Three repeats were used for tetranucleotide, pentanucleotide and hexanucleotide. We used REPuter to analyze forward, palindrome, reverse and complementary sequences with a minimum repeat length of 16 bp and minimum sequence identity greater than 90% [35].

The expansion and contraction of the IR regions were examined with the IRscope (https://irscope.shinyapps.io/irapp/) [36]. The codon usage was analyzed with CodonW [37]. For the nucleotide diversity analysis, complete plastid genome sequences were aligned with MAFFT [32]. A sliding window analysis of window length of 600 bp and step size of 200 bp was used in the DnaSP to estimate the nucleotide diversity values [38]. Structural changes across plastid genomes of Ampelopsideae were analyzed via whole-genome alignment in Mauve 2.4.0 using default parameters [39].

To evaluate the selection pressure on protein-coding genes, we extracted the shared non-redundant genes among species, in which each gene’s CDS-pair of one-by-one species’ combination were extracted and aligned by MAFFT [32]. The rates of synonymous substitutions (Ks) and non-synonymous substitutions (Ka) and Ka/Ks were then calculated by KaKs_Calculator in ParaAT 2.0 [40] using “ParaAT.pl -c 11 -h homologs.txt -n CDS -a PEP -p proc -o OUT -k -f axt -m mafft -v”. The Ka/Ks ratio defines the degree of gene divergence and whether selection pressure is positive (Ka/Ks > 1), purifying (Ka/Ks < 1, particularly if it is less than 0.5), or neutral (Ka/Ks = 1) [41], which is useful for understanding the evolution of protein-coding genes and adaptive developments in species [41, 42].

## Results

### Basic characteristics of plastid genomes of the tribe

**Fig. 1:**
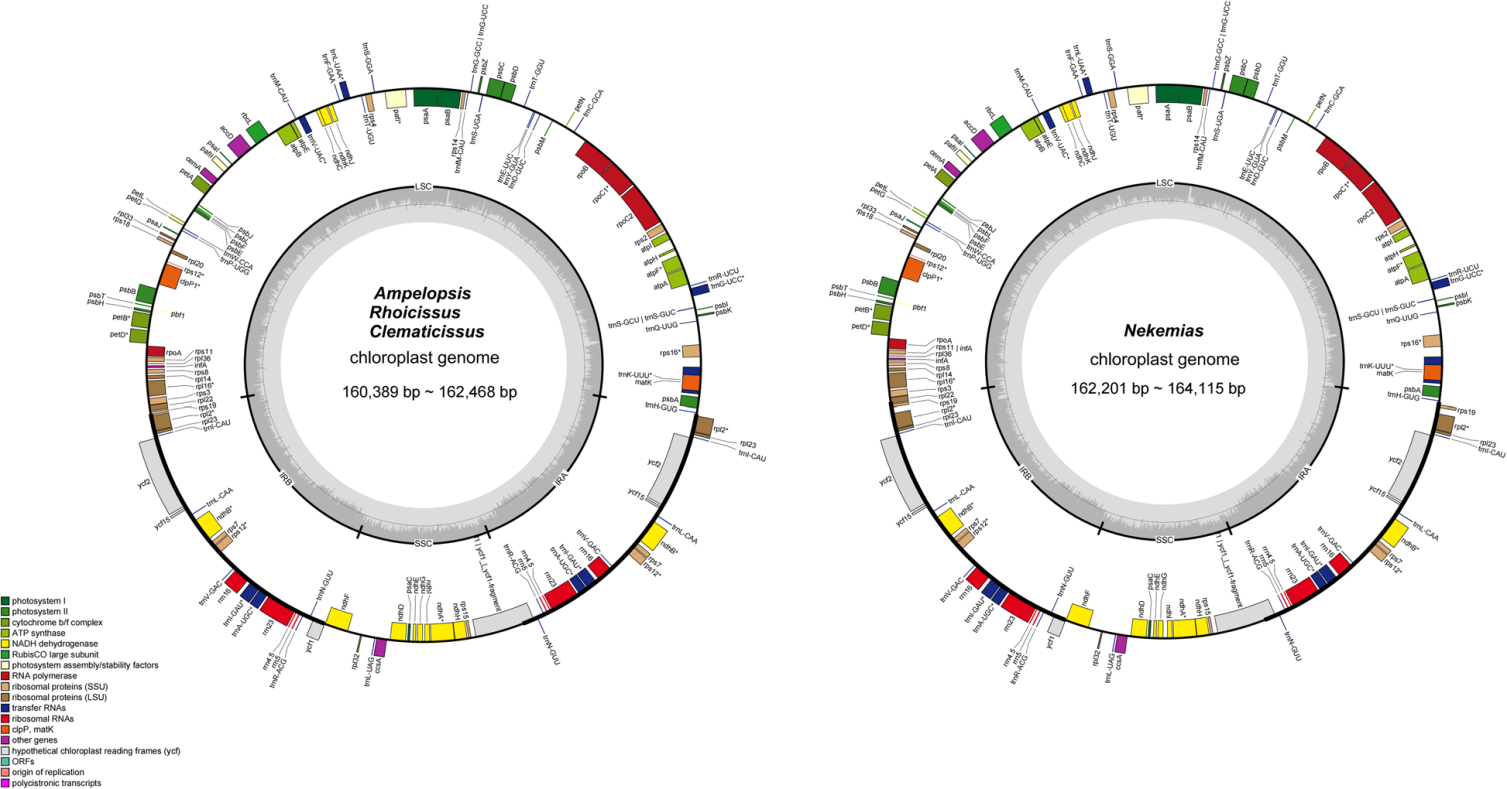
The chloroplast genome maps of Ampelopsideae. Transcriptional directions are represented on the circle’s inside (clockwise) and outside (counterclockwise). Genes are color-coded according to their functional groups

Diagrams of the plastid genomes were presented in Fig. 1. All the plastomes of the tribe show a typical quadripartite structure comprising a LSC region (85,420—93,530 bp) and a SSC region (18,439—21,778 bp) separated by two IR regions (25,689—27,412 bp) (Fig. 1; Table 1). The average GC content of all sequences is ~ 37.4%, including 35.38% for the LSC, 31.85% for the SSC, and 42.6% for the IR region (Table 1). The total number of annotated genes is 133 to 134, comprising 88 to 89 protein-coding, 36 to 37 tRNA, and 8 rRNA genes (Table 1).

**Table 1:** Plastid genome size and gene count in the tribe Ampelopsideae

Species name			Genome size and GC content									
	total		LSC		SSC		IR			Number of genes		
	Length (bp)	G + C (100%)	Length (bp)	G + C (100%)	Length (bp)	G + C(100%)	Length (bp)	G + C(100%)	Gene	CDS	tRNA	rRNA
*Ampelopsis japonica* 2	161,430	37.32	89,627	35.21	18,977	31.85	26,413	42.88	133	88	37	8
*Ampelopsis grandulosa* 3	162,328	37.38	90,270	35.21	18,902	31.81	26,578	42.81	133	88	37	8
*Ampelopsis sinica*	162,468	37.27	90,419	35.16	18,895	31.79	26,577	42.81	133	88	37	8
*Ampelopsis grandulosa* 1	162,460	37.32	90,411	34.58	18,895	31.96	26,577	42.01	133	88	37	8
*Ampelopsis grandulosa* 2	162,348	37.32	90,292	35.21	18,902	31.79	26,577	42.84	133	88	37	8
*Ampelopsis japonica* 1	162,370	37.35	90,312	35.21	18,902	31.79	26,578	42.81	133	88	37	8
*Ampelopsis humulifolia*	161,724	37.33	89,647	35.23	19,032	31.83	26,521	42.84	133	88	37	8
*Ampelopsis heterophylla* 1	162,347	37.32	90,284	35.22	18,897	31.76	26,583	42.84	133	88	37	8
*Ampelopsis heterophylla* 2	162,430	37.30	90,344	35.18	18,920	31.76	26,583	42.79	133	88	37	8
*Ampelopsis bodinieri*	162,368	37.33	90,304	35.21	18,898	31.81	26,583	42.79	133	88	37	8
*Ampelopsis cordata*	162,297	37.36	90,124	35.22	19,069	31.88	26,552	42.83	133	88	37	8
*Rhoicissus tomentosa*	160,884	37.39	88,850	35.07	18,988	31.52	26,523	42.85	133	88	37	8
*Rhoicissus tnidenc*	160,692	37.31	88,659	35.24	18,987	31.54	26,523	42.82	133	88	37	8
*Rhoicissus digitata*	160,760	37.39	88,716	35.18	19,070	31.54	26,487	42.87	133	88	37	8
*Clematicissus simsiana*	162,432	37.44	90,374	34.74	19,082	31.67	26,488	42.91	133	88	37	8
*Clematicissus granulosa*	161,687	37.46	89,567	35.07	19,239	31.72	26,470	42.87	133	88	37	8
*Clematicissus striata* ssp argentina	160,653	37.49	88,612	35.48	19,015	31.83	26,513	42.85	133	88	37	8
*Clematicissus striata*	160,389	37.45	88,324	35.42	19,055	31.83	26,505	42.86	134	89	37	8
*Clematicissus opaca*	161,352	37.31	89,085	35.15	19,091	31.67	26,588	42.81	134	89	37	8
*Nekemias megalophylla*	162,201	37.32	88,868	35.27	18,947	31.82	27,193	42.45	134	89	37	8
*Nekemias hypoglauca*	163,310	37.35	89,625	35.36	19,063	31.69	27,311	42.45	134	89	37	8
*Nekemias cantoniensis* 1	162,860	37.32	89,235	35.37	19,083	31.69	27,271	42.49	134	89	37	8
*Nekemias cantoniensis* 2	163,056	37.37	89,165	35.37	19,067	31.71	27,412	42.39	134	89	37	8
*Nekemias rubifolia*	162,983	37.35	89,358	35.31	19,077	31.67	27,274	42.49	134	89	37	8
*Nekemias grossedentata* 1	162,165	37.41	89,262	35.35	18,439	31.82	27,232	42.51	134	89	37	8
*Nekemias celebica*	162,811	37.36	89,155	35.44	19,066	31.70	27,295	42.47	134	89	37	8
*Nekemias grossedentata* 2	162,566	37.38	89,331	35.32	19,051	31.71	27,092	42.63	134	89	37	8
*Nekemias grossedentata* 3	162,590	37.38	89,377	35.30	19,071	31.69	27,071	42.65	134	89	37	8
*Nekemias cantoniensis* 3	162,739	37.36	88,966	35.27	19,055	31.72	27,359	42.41	133	89	36	8
*Nekemias arborea*	164,115	37.31	90,959	34.93	21,778	32.22	25,689	42.95	134	89	37	8
*Parthenocissus semicordata*	161,828	37.41	89,881	35.36	19,153	31.65	26,397	42.91	133	88	37	8
*Parthenocissus quinquefolia*	161,200	37.48	89,298	35.51	18,960	31.83	26,471	42.86	133	88	37	8
*Cissus antarctica*	162,215	37.53	90,063	35.34	19,092	31.78	26,530	42.95	133	88	37	8
*Cissus acrantha*	163,707	37.65	93,530	35.76	19,082	31.91	26,527	43.11	133	88	37	8
*Cayratia lineats*	160,155	37.72	88,427	35.84	19,050	32.17	26,339	42.88	133	88	37	8
*Pseudocayratia dichromocarpa*	157,486	37.55	85,420	35.58	18,850	31.52	26,608	42.85	133	88	37	8

Of the 18 duplicated genes in the IR, seven are protein-coding, seven are tRNA, and four are rRNA genes. We observed gene duplication and loss in the plastid genes of some species (Table 2). For example, copies of the *rps19* gene were found in all genera of *Nekemias.* Additionally, *Nekemias arborea* (L.) J. Wen & Boggan has a deletion of the *ycf1*. We also found pseudogenes in our assembled data, such as *rps19* pseudogene (ψ*rps19*), *ycf1* pseudogene (ψ*ycf1*), and *ndhI* pseudogene (ψ*ndhI*) (Table 2).

**Table 2:** Plastid gene types and functions in the tribe Ampelopsideae

Category	Gene group	Gene name
Self-replication	Ribosomal RNA genes	*rrn23, rrn16, rrn5, rrn4.5*
	Transfer RNA genes	*trnA-UGC, trnC-GCA, trnD-GUC, trnE-UUC, trnF-GAA, trnfM-CAU, trnG-UCC, trnG-GCC, trnH-GUG, trnI-CAU, trnI-GAU, trnK-UUU, trnL-CAA, trnL-UAA, trnL-UAG, trnM-CAU, trnN-GUU, trnP-UGG, trnQ-UUG, trnR-ACG, trnR-UCU, trnS-GCU, trnS-GGA, trnS-UGA, trnT-GGU, trnT-UGU, trnV-GAC, trnV-UAC, trnW-CCA, trnY-GUA*
	Small subunit of ribosome	*rps11, rps12, rps14, rps15, rps16, rps18, rps19, rps2, rps3, rps4, rps7,rps8,*
	Large subunit of ribosome	*rpl14, rpl16, rpl2, rpl20, rpl22, rpl23,rpl32, rpl33, rpl36*
	DNA-dependent RNA polymerase	*rpoA, rpoB, rpoC1, rpoC2*
	Translational initiation factor	*infA*
Genes for photosynthesis	Subunits of photosystem I	*psaA, psaB, psaC, psaI, psaJ*
	Subunits of photosystem II	*psbA, psbB, psbC, psbD, psbE, psbF, psbH, psbI, psbJ, psbK, psbL, psbM, psbT, psbZ*
	Subunits of cytochrome	*petA, petB, petD, petG, petL, petN*
	Subunits of ATP synthase	*atpA, atpB, atpE, atpF, atpH, atpI*
	Large subunit of Rubisco	*rbcL*
	Subunits of NADH dehydrogenase	*ndhA, ndhB, ndhC, ndhD, ndhE, ndhF, ndhG, ndhH, ndhI, ndhJ, ndhK*
Other genes	Maturase	*matK*
	Envelope membrane protein	*cemA*
	Subunit of acetyl-CoA	*accD*
	C-type cytochrome synthesis gene	*ccsA*
	Protease	*clpP1*
	Component of TIC complex	*ycf1*
	Pseudogene	*ψrps19, ψycf1, ψndhI*

### Phylogenetic relationships

**Fig. 2:**
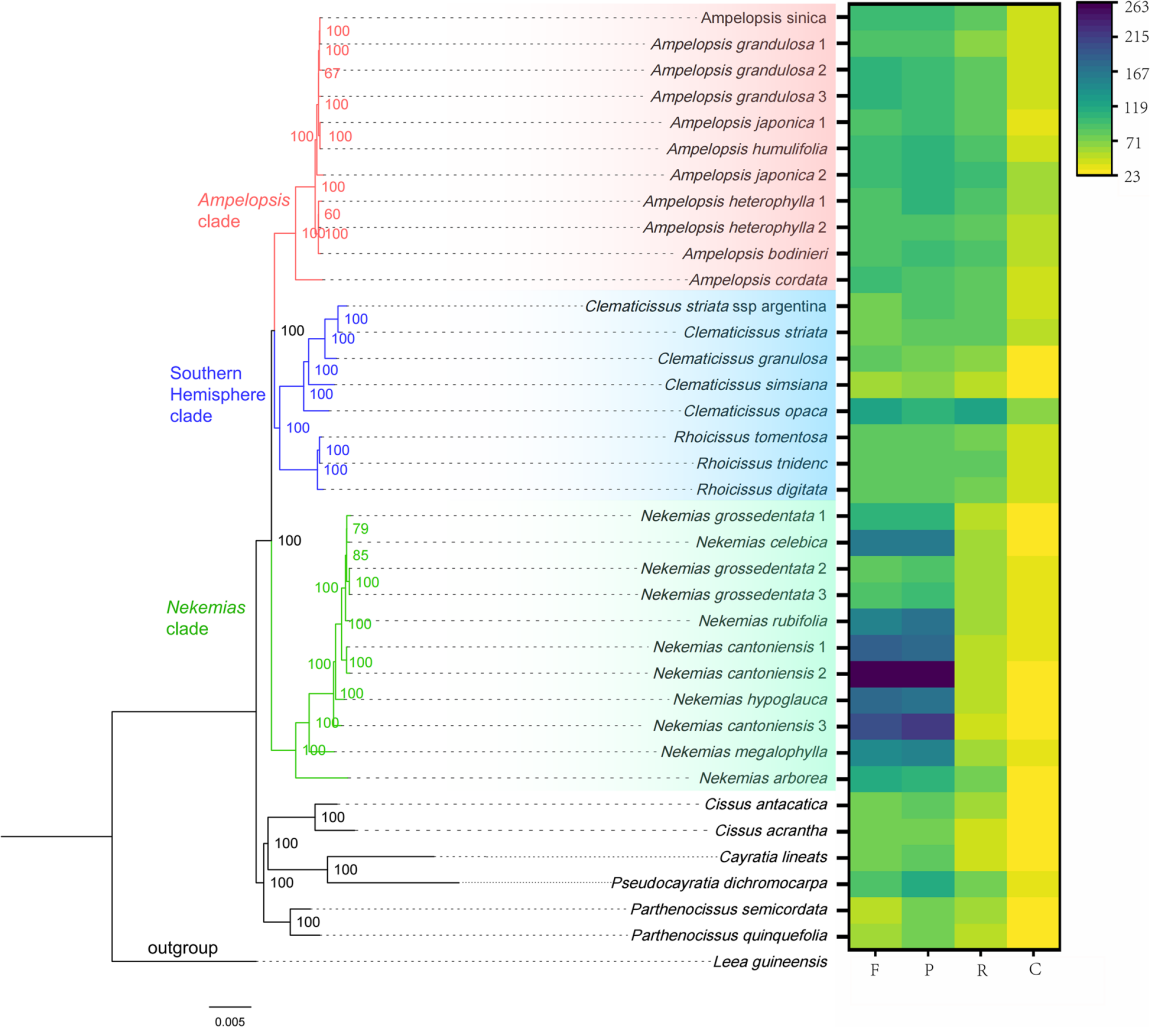
A ML tree of Ampelopsideae inferred from complete chloroplast genomes. Numbers near nodes represent bootstrap support values. The heat map shows different repeat sequence types and numbers for each taxon

A ML tree was reconstructed based on all the plastid genome data (Fig. 2). The plastid phylogeny of Ampelopsideae has a high level of resolution as most relationships supported with strong to medium support values (BS > 75%). Three main clades were recognized within the tribe, corresponding to *Ampelopsis*, *Nekemias*, and the Southern Hemisphere clade, respectively, and all received 100% bootstrap values (Fig. 2). Within the *Ampelopsis* clade, the North American species *Ampelopsis cordata* Michx. represents as the first diverged lineage, sister to the remaining members from East Asia (Fig. 2). The North American species from *Nekemias* serves as the first divergent lineage sister to the East Asian group (Fig. 2). For taxa from the Southern Hemisphere, the African *Rhoicissus* is sister to the expanded *Clematicissus* with taxa from the South American species forming a clade sister to the Australian species (Fig. 2).

### Plastome structure and length variation

**Fig. 3:**
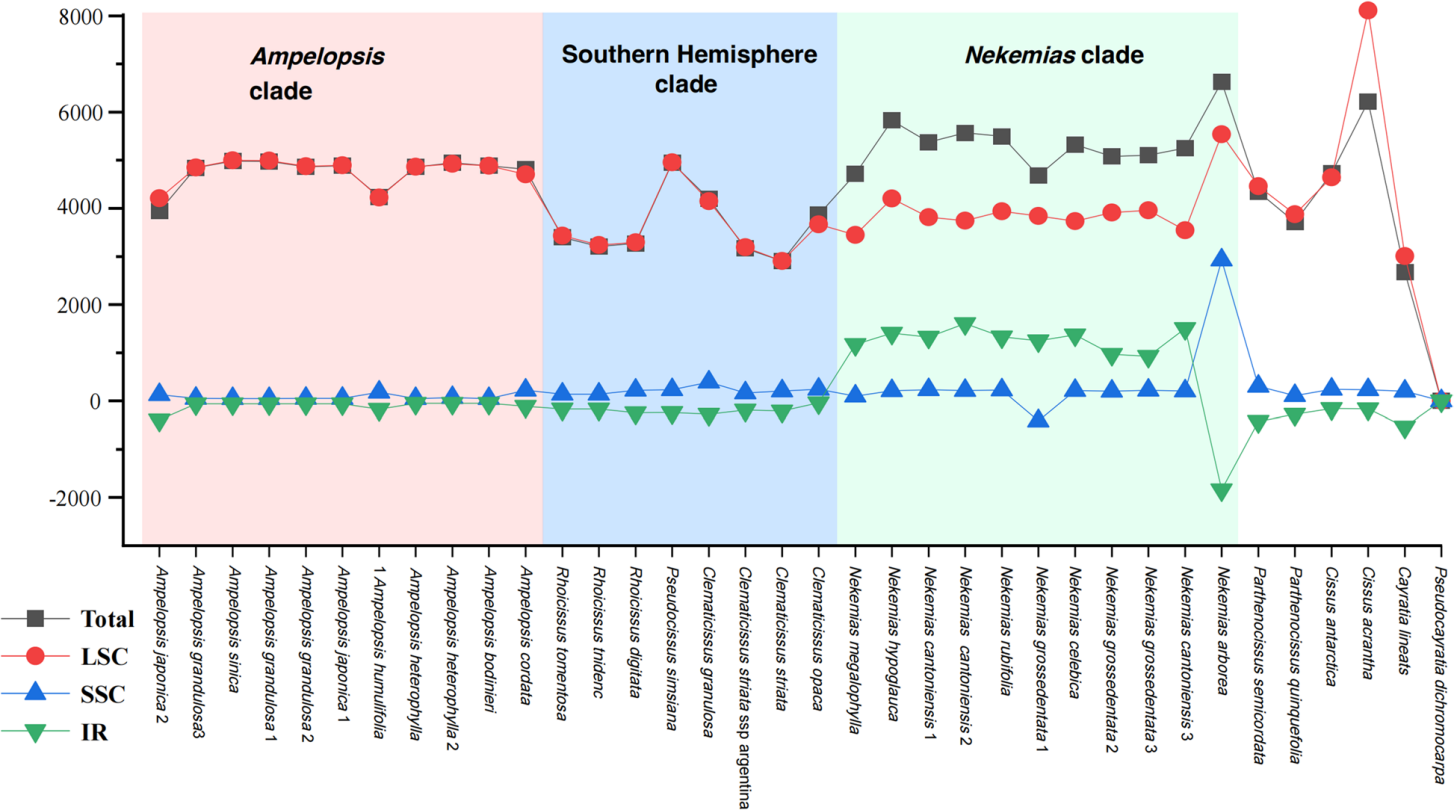
Length variation in the plastid genomes of Ampelopsideae. The y-axis values are minus data for the smallest genome of *Pseudocayratia dichromocarpa*

The variation in total length of the chloroplast genomes and the sizes of each region among the three clades within the tribe are presented in Fig. 3. The plastid sequences of the tribe exhibit large variation in length, ranging from 160,692 to 163,219 bp (Table 1). *Nekemias* shows the longest average length of 162,854 bp within the tribe, ranging from 162,165 to 164,115 bp, and *Ampelopsis* has a close average length of 162,233 bp, ranging from 161,430 to 162,468 bp (Table 1). In contrast, the Southern Hemisphere lineage exhibits the shortest average length of 161,106 bp, ranging from 160,389 to 162,432 bp (Table 1). The LSC region of *Ampelopsis* is the largest, with an average size of 90,184 bp (ranging from 89,627 bp to 90,419 bp), and *Nekemias* shows the next largest average length as 89,391 bp (ranging from 88,868 bp to 90,959 bp) (Table 1). The IR region of *Nekemias* has an average size of 27,109 bp (ranging from 25,689 bp to 27,412 bp), while the other two clades have similar smaller average size (Table 1). The SSC region is relatively similar among the three clades, ranging from 18,895 to 21,778 bp.

**Fig. 4:**
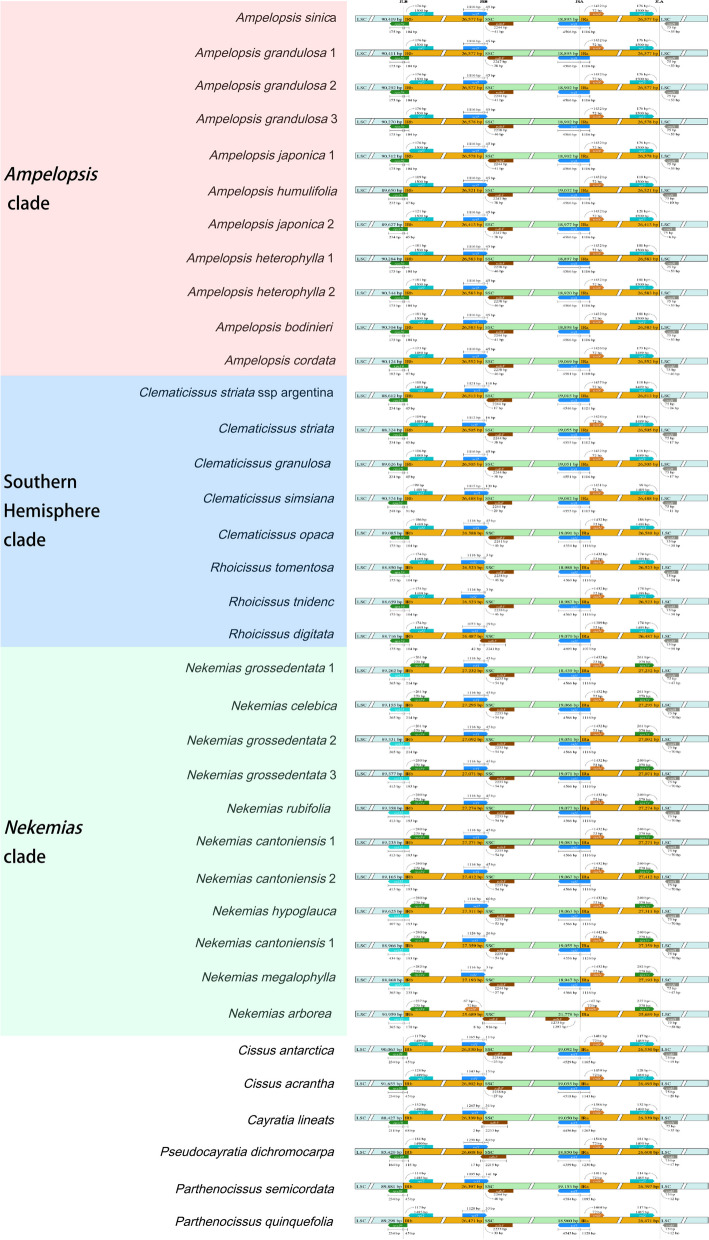
Comparison of the gene order and IR/SC junction sites in Ampelopsideae plastomes (covering the three lineages of the tribe). The number of base pairs indicates the distance between the end of the gene and the junction site. Boxes above and below the regions represent genes transcribed in the forward and reverse DNA strands, respectively

The plastid genomes of Ampelopsideae show no significant differences in the boundaries of the IR and SSC regions, except for *N. arborea*, where the *ycf1* pseudogene and *ycf1* gene are not found at the IRb-SSC boundary and the IRa-SSC boundary, respectively (Fig. 4). Additionally, in *Rhoicissus digitata* (L.f.) Gilg & Brandt, the *ndhF* gene spanned 42 bp across the JSB (IRb-SSC boundary) (Fig. 4). In the LSC and IR boundaries, *Ampelopsis* and the Southern Hemisphere taxa show the *rps19* gene spanning the JLB (LSC-IRb boundary), the *rpl22* gene located near the JLB in the LSC region, and the *rpl2* gene on the left side of JLA (Fig. 4). In contrast, *Nekemias* species exhibit different pattern, with the *rpl22* gene presented on the JLB, the *rps19* gene located in the IR region to the right of JLB, and the *rps19* gene on the left side of JLA (Fig. 4).

No gene rearrangements were found in the plastid genome of each genus in the Ampelopsideae (Fig. S1). Furthermore, the gene arrangement of the tribe was found to be similar to other species of Vitaceae (Fig. S1). Nucleotide diversity values were calculated for Ampelopsideae, with the highest nucleotide diversity observed in the SSC region (0.0423) and those in the IR region were less than 0.003 (Fig. S2).

### Repetitive sequences and SSR

Among the four different types of repetitive sequences, the number of forward repeats (F-type) and palindromic repeats (P-type) is higher than the number of complement repeats (Fig. 2). *N. cantoniensis* 2 has the largest total number of 602 repetitive sequences (Fig. 2). However, the number of the two types varied widely among species, with the largest number found from *N. cantoniensis* 2, including 261 P-types and 263 F-types (Fig. 2). In addition, the number of duplication of both F-type and P-type was relatively high in *Nekemias*, while the number of reverse repeats (R-type) was slightly lower than that of other genera (Fig. 2).

**Fig. 5:**
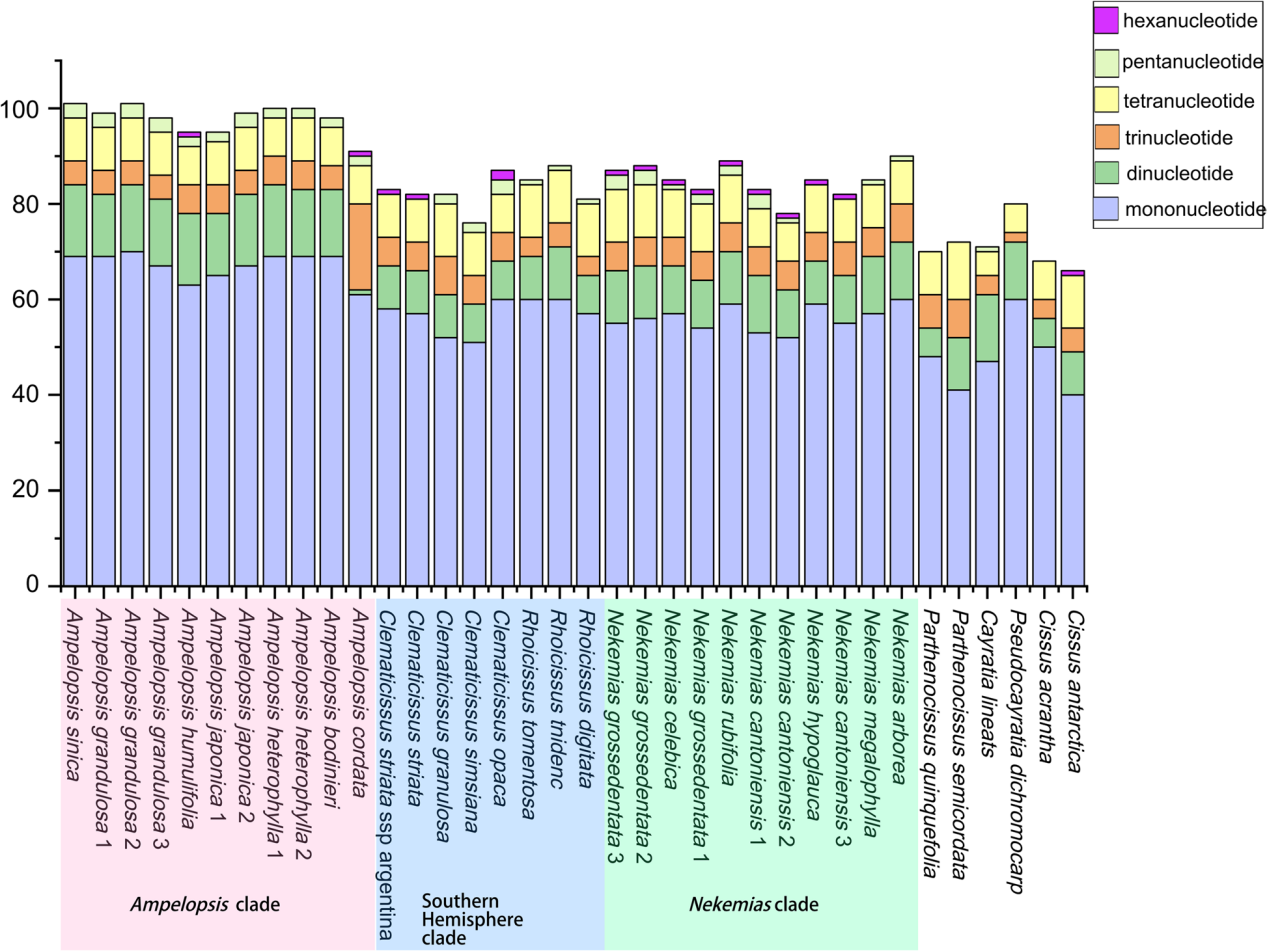
SSR types in Ampelopsideae plastomes

A total of 27 different types of SSRs were found in the tribe (Fig. 5). A/T and AT/AT repetitions account for most of them, and A/T, AT/AT, AAT/ATT and AAAT/ATTT are the simple repetition type common to all species. Some simple repeat types such as ACT/AGT, AAAC/GTTT, and AATT/AATT occur once in some species, while AAAG/CTTT and AATC/ATTG are missing in some species (Table S2). The number and type of SSRs in the SC and IR regions are different. Most of the SSRs were found in the SC region, with more than 75% of the total SSRs found in the LSC region.

### Codon usage

**Fig. 6:**
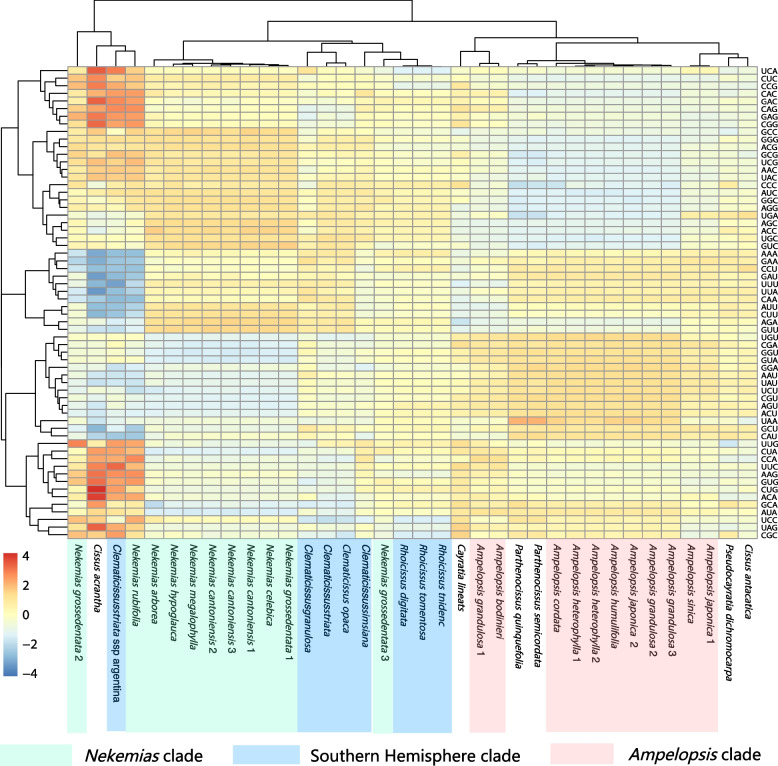
The heat map of codon usage bias in the chloroplast genomes of Ampelopsideae. The color depth represents the Euclidean distance

The relative synonymous codon usage (RSCU) frequency was calculated using 88 protein-coding sequences from the plastid genome. Among all amino acids, Leu is the mostly used codons and Cys is the least one (Fig. S3). Compared synonymous codon usage analysis (Fig. S3, Fig. 6) discovered that RSCU value of 30 to 31 codons is greater than 1 (Fig. 6). Met and Trp have no biased usage (RSCU = 1). Among the codons with RSCU > 1 in the Ampelopsideae, only the Leu codon (UUG) is G-ending, and the other 29 to30 codons are A or U-ending. AGA, which encodes the Arg amino acid, is the most preferred codon (minimum value of preference index > 1.756), while CGC is the one with the lowest preference index (maximum value of preference index < 0.377). In the cluster analysis using codon preference, the clustering tree was largely grouped into three major blocks, corresponding to the three clades of the tribe (Fig. 6).

### Selective pressure evaluation

**Fig. 7:**
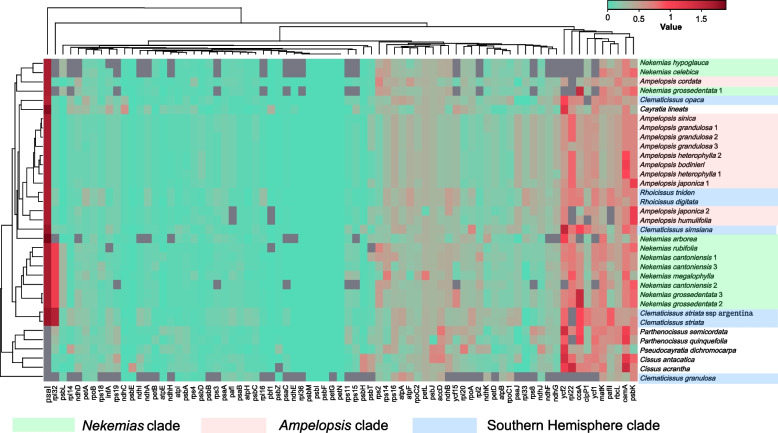
The heat map showing pairwise Ka/Ks ratios between concatenated single-copy coding sequences among Ampelopsideae plastid genomes

We compiled a data matrix comprising the Ka/Ks values of 54 gene pairs across a total of 35 species (Fig. 7). Excluding genes for which the Ka/Ks values could not be determined, our analysis yielded a total of 2,240 gene loci with Ka/Ks less than 0.5, 218 gene loci with Ka/Ks greater than 0.5 but less than 1, and only 58 gene loci exhibited with Ka/Ks greater than 1 (Fig. 7). We detected that 34 genes in Ampelopsideae exhibited Ka/Ks values close to or equal to 0 (Fig. 7). Additionally, we observed positive selection acting on the psaI gene across the whole Ampelopsideae, while *ccsA*, *cemA*, *psbK*, *rpl32*, and *ycf2* exhibit Ka/Ks greater than 1 in some species. The *rpl32* gene is under positive selection in almost all members of *Nekemias* and some *Clematicissus* species from the Southern Hemisphere (Fig. 7).

## Disussions

Plastid genomes tend to be stable and conserved in plants [43, 44]. Our findings (Table 1) suggested that Ampelopsideae plastomes are largely consistent with previous reports in terms of structure, gene number, RNA and protein-coding genes, and GC content [45–48]. The GC content was found to be higher in the IR region than the SC region, likely due to the agglomeration of four rRNA genes in the IR region [49]. Conversely, the SSC region exhibited a lower GC content than that of the LSC region, which could be attributed to ndh gene clustering in the SSC region.

This study reconstructed well-supported phylogenetic relationships of the Ampelopsideae based on the plastid genomic sequences, which represented the first phylogeny of the tribe based on a broad sampling of plastid genomes (Fig. 2). Our study was largely consistent with previous results [2, 7, 11]. The plastid genomes provided robust support for the recently resurrected *Nekemias* as a distinct monophyletic genus, separate from *Ampelopsis* [7–9, 50], suggesting *Nekemias* as the first diverged lineage within the tribe, sister to a clade including *Ampelopsis* and taxa from the Southern Hemisphere, a backbone relationship of the tribe congruent with those reported by recent studies [1, 7, 11, 50, 51]. Furthermore, our data improved resolution throughout the tribe compared with previous studies, with almost all nodes being strongly supported (Fig. 2).

Ampelopsideae exhibits variations in size across the plastid genome as well as within the LSC and IR regions consistent with the phylogenetic relationships (Fig. 3). Most species in Ampelopsideae show minimal variation in the SSC (Fig. 3), indicating that the impact on plastid genome size is primarily driven by changes in the LSC and IR regions. The length variation of the IR regions is commonly found in the plastid genomes of angiosperms, which often leads to changes the number of genes in various plant lineages [43, 48, 52–56]. Compared with the other two clades, the *Nekemias* clade shows a distinguishable expansion in the IR region (Fig. 3). *Nekemias* has a complete duplicated copy of the *rps19* gene in the IRa region and the *rpl22* gene is incorporated more often into the IRb region (Fig. 4). Correspondingly, the LSC region is reduced in *Nekemias* due to the *rps19* gene is assigned to the IR region. The IR expansion resulted from the generation of a pseudo-copy or functional gene copy of a single-copy gene with transferring from LSC or SSC to IRs [48, 56, 57]. Previous reports have shown that in monocots that IR expansion occurs at the IRa/LSC boundary, resulting in a duplicated copy of the *trnH-GUG* gene adjacent to *rps19* at the IRb/LSC boundary [17]. The *rps*19 protein is a component of the 40S ribosomal subunit and belongs to a family of ribosomal proteins restricted to eukaryotes and archaea [58]. Although the evolutionary significance is unclear for the increased copy of the *rps19* in *Nekemias*, we illustrated an interesting case of the independent duplication of *rps19* in the IR region of *Nekemias* within the Ampelopsideae.

On the other hand, the overall length of the plastid genome and LSC region for the Southern Hemisphere clade is smaller than those of the other two clades (Fig. 3). Although the LSC region has expanded into the IR region, it did not ultimately result in an overall expansion of the LSC region. This suggests that the expansion of the LSC region into the IR region is not the primary cause of the size variations in different regions for the Southern Hemisphere clade. Because there is no gene loss in the LSC region, the size variations in this clade are probably due to partial deletions and insertions in intergenic spacer regions. Furthermore, the expansion and contraction of the SSC region specific to *N. arborea* and *Nekemias grossedentata* 1 (Hand.-Mazz.) J. Wen & Z.L. Nie (Fig. 3) may be the result of interactions with the IR region and the loss of intergenic region segments within the SSC region.

Repetitive sequences play diverse roles in cellular processes, including gene evolution, gene expression, mRNA stabilization, gene organization, gene mobility, cellular immunity against foreign genes, and even gene engineering in prokaryotes and eukaryotes [59–65]. The F-type and P-type are more abundant than R-type and C-type, a pattern consistent with previous findings in other plant taxa [66, 67]. These repeat sequences are pivotal for genome reconfiguration and have been associated with numerous insertions and deletions [57]. The prevalence of such repeats could enhance nucleotide diversity [68], providing a basis for evolutionary and population genetic studies [69]. This could signify the important roles that F-type and P-type in genetic recombination, DNA repair, and replication fidelity. The SSRs in Ampelopsideae, particularly the highly abundant in poly-A and T motifs (Table S2), are potential molecular markers due to their high polymorphism and mutation rates [25, 70–75].

The types and content of repetitive sequences in the Ampelopsideae display variation among clades (Fig. 2), indicating that they may have undergone distinct evolutionary trajectories and adapted to different ecological niches. Notably, the *Nekemias* shows a higher abundance of F-type and P-type repeats (Fig. 2), which may suggest that the genus possesses genome characteristics distinct from both *Ampelopsis* and the Southern Hemisphere taxa. Interestingly, despite both *Nekemias* and *Ampelopsis* primarily distributed in East Asia, *Ampelopsis* shared a lower number of F- and P-type but relatively higher number of R-type and C-type repeats similar to that of the Southern Hemisphere taxa (Fig. 2). *Ampelopsis* and *Nekemias* have a similar distribution and habitats mainly in East Asia, these differences of repeat types likely reflect the distinct evolutionary history and ecological adaptations to local niches, which may have arisen in response to different selective pressures and environmental conditions [1, 2]. Overall, the identification and characterization of repetitive sequences in different taxa of the Ampelopsideae provide valuable insights into understanding their evolutionary diversification and ecological adaptation in East Asia.

We found that specific codons are more frequently used in the nucleotide sequences of protein-coding genes in the plastid genome of the Ampelopsideae than other synonymous codons (Fig. S3), consistent with previous reports [25]. All preferred synonymous codons (RSCU > 1) end with A or U, which may contribute to the bias towards A/T bases throughout the genome. In contrast, codons ending with C, such as CGC (Arg), UGC (Cys), CAC (His), and AGC (Ser), have relatively low RSCU values (Fig. S3). RSCU can affect gene expression by regulating the accuracy and efficiency of gene translation, with stronger RSCU leading to higher gene expression levels[76–78]. Known codon usage patterns can also be used to predict the expression and function of unknown genes [47]. Synonymous codons with RSCU > 1 can be used as indicators for detecting the expression levels of hypothetical genes or open reading frames and can be used in designing primers, introducing point mutations, and other breeding research [76–78].

The evolutionary rates and patterns of SCUB in plant plastid genomes display unique characteristics compared to mitochondrial and nuclear genomes [79]. SCUB is shaped by various factors including directional mutation pressure, natural selection, *trnA* abundance, strand-specific mutation bias, gene expression levels, and gene length [80–83]. These determinants have been instrumental in explaining codon usage variations both within and between species [84]. Most of the phylogenetically related groups within the Ampelopsideae were clustered together, reflecting the similarity in codon usage bias among closely related species (Fig. 6), with smaller Euclidean distances between species indicating closer genetic relationships [85]. Our results suggest that species from the same genus or subclade face similar directional mutation pressure and natural selection, resulting in similar codon usage bias.

Regarding genetic diversity, our analysis revealed that the SC regions of the Ampelopsideae exhibits doubled nucleotide diversity in comparison with the IR regions (Fig. S2). This discrepancy could be attributed to the high conservation of crucial genes found within the IR regions [86–90]. A plausible explanation for this observation is the presence of essential housekeeping genes, such as structural ribosomal RNA genes (*rrn4.5*, *rrn23*, and *rrn16*), which are highly conserved even in organisms with shorter IRs that primarily contain rRNA genes and limited intergenic spacers, as observed in certain algae [90]. Notably, the highest nucleotide diversity was found at the boundary between the IRs and SSC regions (Fig. S2), which might be caused by fine rearrangements during the contraction and expansion of the IR region's boundaries.

Purifying selection, one of the most prevalent forms of natural selection, constantly removes deleterious mutations in populations [91]. The low Ka/Ks ratios observed at the chloroplast genome within the tribe indicate that most genes are subject to purifying selection to retain conserved functions (Fig. 7). Positive selection has been found in genes related to photosynthesis in some weakly light-adapted aquatic plants [91]. In most cases, genes related to specific environments are typically assumed to be under positive selection [92]. The *psaI* gene, encoding a reaction protein complex in Photosystem I of plant chloroplasts, plays a crucial role in photosynthetic pigment reactions [93]. The *psaI* gene may be a candidate gene for adaptive evolution in response to the specific growth environment of Ampelopsideae species since their small and creeping growth habit under forestry push them in competition for sunlight with taller trees or shrubs.

## Conclusions

This study demonstrated the conservation of genome size, gene number, and GC content within the Ampelopsideae, with no major gene rearrangements observed. But our results also indicated that plastomes wihin the tribe vary among three lineages in genome length, expansion or contraction of the inverted repeat region, codon usage bias, and repeat sequences, probably due to different environmental selection pressures and evolutionary histories. Furthermore, some specific genes are under positive selection, such as *psaI* and *rpl32*, suggesting that they are significant in the Ampelopsideae evolution. Building on the solid phylogenetic and evolutionary framework established here, future studies with even greater taxonomic and genomic sampling may contribute to a better understanding of the diversification patterns in Ampelopsideae in relation to climatic, biogeographic, and ecological factors.

### Supplementary Information

**Supplementary Material 1.** 

**Supplementary Material 2.** 

**Supplementary Material 3.** 

**Supplementary Material 4.** 

**Supplementary Material 5.**

## Data Availability

Chloroplast data for all species in this paper have been uploaded to NCBI and collection information and name coding with species samples are recorded in Supplementary Table 1. Additionally, it includes accession numbers of the reference species downloaded from NCBI.
