# Supplementary material for: Plastid genome data provide new insights into the dynamic evolution of the tribe Ampelopsideae (Vitaceae)
Source: BMC Genomics. 2024 Mar 5;25:247. doi: 10.1186/s12864-024-10149-w (PMC10916268; doi:10.1186/s12864-024-10149-w)
Supplement: Supplementary file 3 — Supplementary Material 3. [file 12864_2024_10149_MOESM3_ESM.docx]

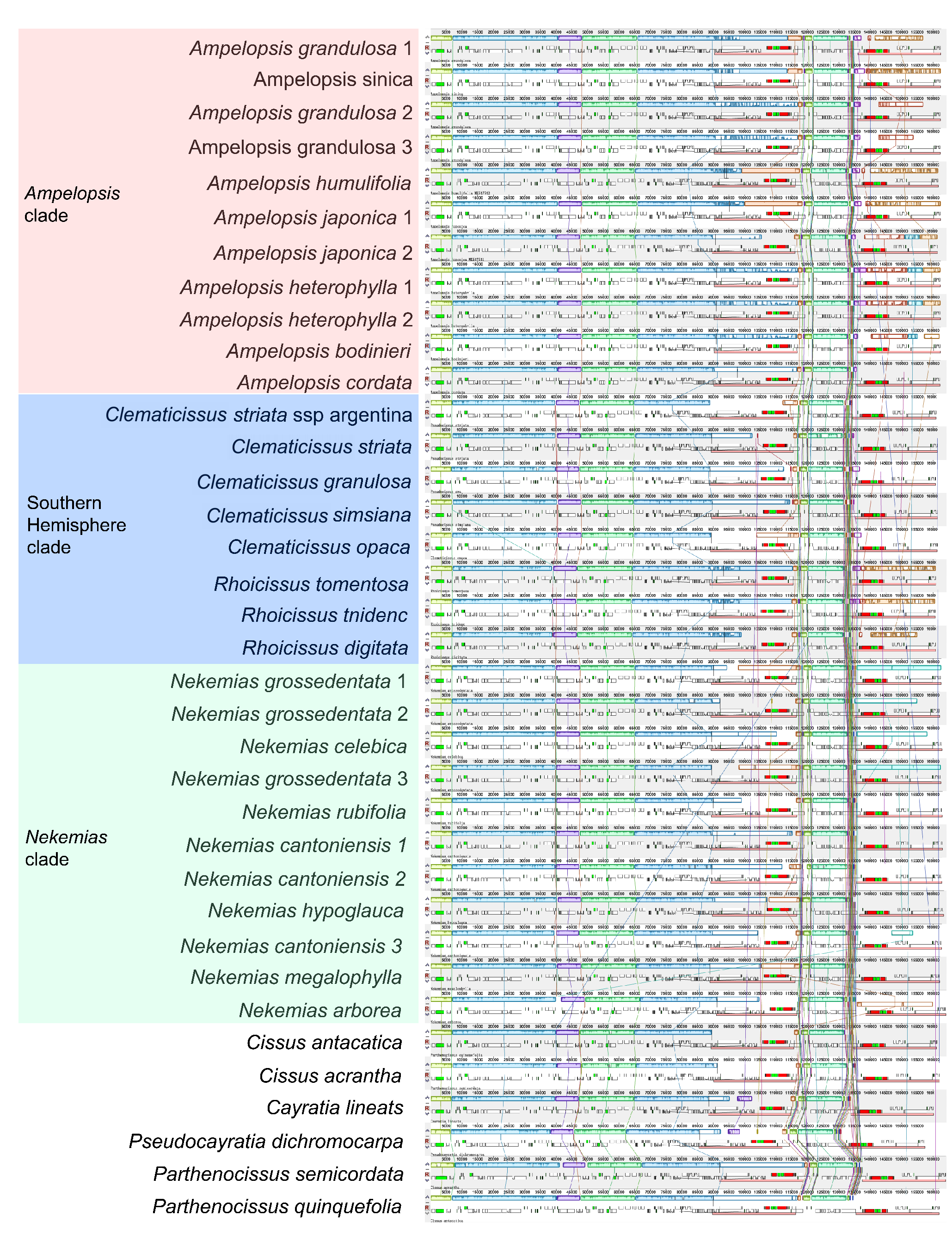


**Figure S1.** Collinearity analysis of the tribe Ampelopsideae. The same color block represents identical or similar base fragments, and the lines connect blocks of different species.
