# Supplementary figures and images for: Plastid genome data provide new insights into the dynamic evolution of the tribe Ampelopsideae (Vitaceae)
Source: BMC Genomics. 2024 Mar 5;25:247. doi: 10.1186/s12864-024-10149-w (PMC10916268; doi:10.1186/s12864-024-10149-w)

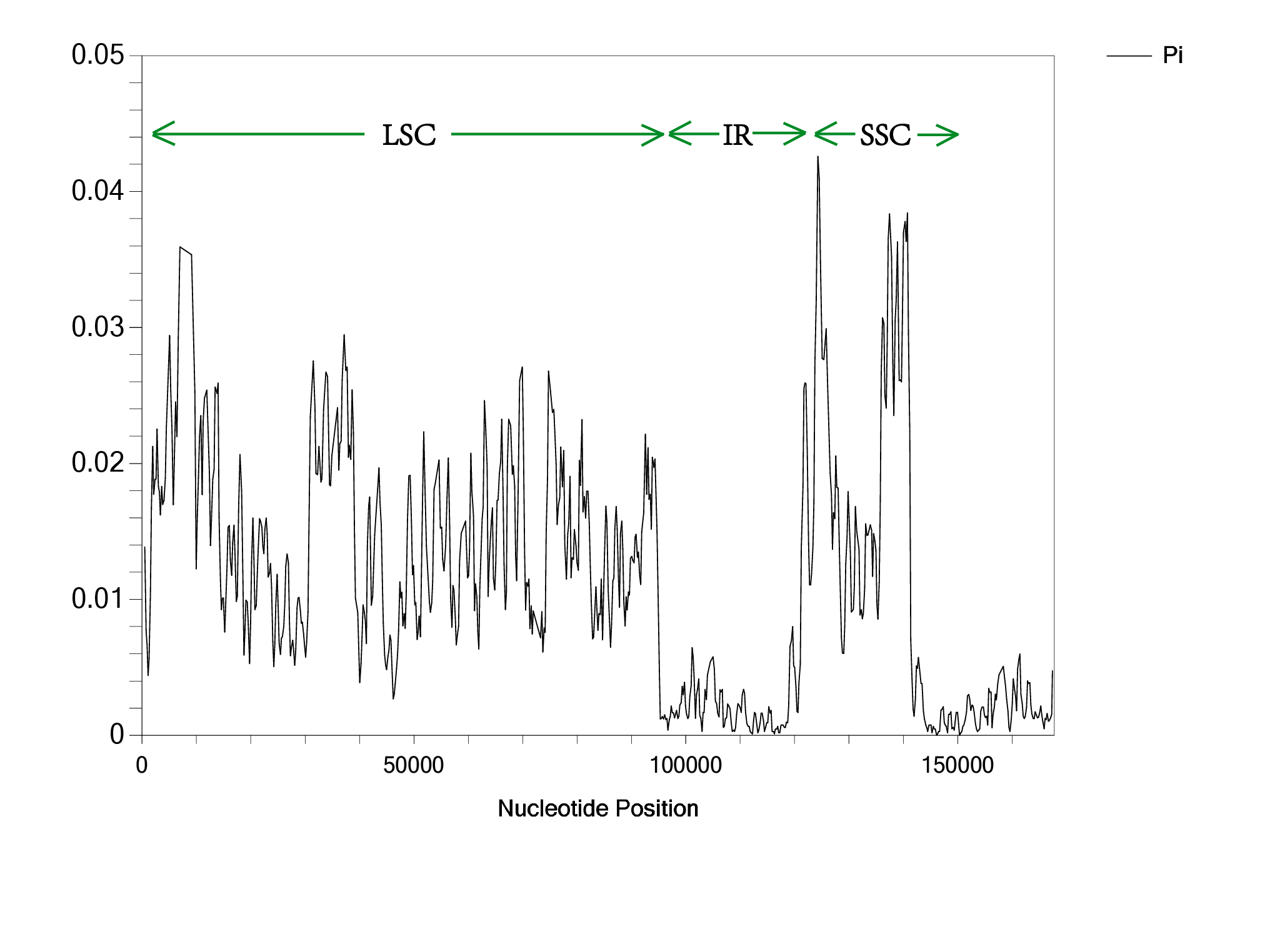


**Figure S2**. Nucleotide diversity (pi) of Ampelopsideae.

Supplement: Supplementary file 4 — Supplementary Material 4. [file 12864_2024_10149_MOESM4_ESM.docx]
