# Supplementary material for: Plastid genome data provide new insights into the dynamic evolution of the tribe Ampelopsideae (Vitaceae)
Source: BMC Genomics. 2024 Mar 5;25:247. doi: 10.1186/s12864-024-10149-w (PMC10916268; doi:10.1186/s12864-024-10149-w)
Supplement: Supplementary file 5 — Supplementary Material 5. [file 12864_2024_10149_MOESM5_ESM.docx]

**Figure S3.** RSCU of Ampelopsideae. The deeper the color depth, the higher the RSCU value.
